# The complete chloroplast genome of *Curcuma longa L.* (Zingiberaceae)

**DOI:** 10.1080/23802359.2019.1666665

**Published:** 2019-09-18

**Authors:** Jie Luo, Mingzhi Li, Guo Yang

**Affiliations:** a Academy of Yuanpei, Shaoxing University, Shaoxing, China;; b Independent Researcher, Guangzhou, China;; c Academy of Life Science, Shaoxing University, Shaoxing, China

**Keywords:** *Curcuma longa*, complete chloroplast genome, phylogenetic analysis

## Abstract

*Curcuma longa L.* is a widely distributed medicinal plant in Southeast Asia, especially in China. In this study, we reported and characterized the complete chloroplast genome sequence of *C. longa.* The chloroplast genome was determined to be 162,255 bp in length. It contained large single-copy (LSC) and small single-copy (SSC) regions of 86,975 bp and 15,776 bp, respectively, which were separated by a pair of 29,752 bp inverted repeat (IR) regions. The genome is predicted to contain 133 genes, including 87 protein-coding genes, 38 tRNA genes, and eight rRNA genes. The overall GC content of the genome is 36.2%. A phylogenetic tree reconstructed by 23 chloroplast genomes reveals that *C. longa* is mostly related to *Curcuma roscoeana*, with bootstrap support values of 100%.

The genus *Curcuma* comprises more than 50 species and distributed widely in Southeast Asia (Fang et al. 1999). There are about five species of this genus in China. *Curcuma longa* is a widely distributed medicinal plant in South and Southwest China. But, to date, there is still no complete cp genome was characterized for *C. longa*. Here, we characterized the complete chloroplast (cp) genome sequence of *C. longa*; the first complete chloroplast genome sequence from the genus *C. longa*, based on the genome skimming sequencing data.

The total genomic DNA was extracted from the fresh leaves of *C. longa.* (South China Botanical Garden, N23°10′54.84″, E113°21′46.61″) using the Plant DNA Mini Kit (Genepioneer, Nanjing, China). Specimens were stored in the Herbarium of Shaoxing University (accession number: SXU-20190415JH01). The whole genome sequencing was conducted by Nanjing Genepioneer Biotechnologies Inc. (Nanjing, China) on the Illumina Hiseq platform. The filtered sequences were assembled using the program SPAdes assembler 3.10.0 (Bankevich et al. 2012). Annotation was performed using the DOGMA (Wyman et al. 2004) and BLAST searches.

The cp genome of *C. longa* was determined to comprise a 162,255 bp double stranded, circular DNA (NCBI acc. no. MK965541), and it contained two inverted repeat (IR) regions of 29,752 bp, separated by large single-copy (LSC) and small single-copy (SSC) regions of 86,975 bp and 15,776 bp, respectively. The cp genome was predicted to contain 133 genes, including 87 protein-coding genes, 38 tRNA genes, and eight rRNA genes. Seven protein-coding genes, eight tRNA genes, and four rRNA genes were duplicated in IR regions. Nineteen genes contained one intron and two genes (clpP and ycf3) contained two introns. The overall GC content of *C. longa* cp genome is 36.2% and the corresponding values in LSC, SSC, and IR regions are 34.0%, 29.5%, and 41.1%, respectively.

To investigate its taxonomic status, alignment was performed on the 23 chloroplast genome sequences using MAFFT v7.307 (Katoh and Standley 2013), and a maximum-likelihood (ML) tree (*Sabal domingensis* and *Typha latifolia* were used as the outgroup) was constructed using FastTree version 2.1.10 (Price et al. 2010). As expected *C. longa* is mostly related to *Curcuma roscoeana*, with bootstrap support values of 100% (Figure 1), witch in agreement with previously published phylogenetic studies (Sass et al. 2016). The complete cp genome sequence of *C. longa* will provide a useful resource for the conservation genetics of this species as well as for the phylogenetic studies of Zingiberaceae.

**Figure 1.**
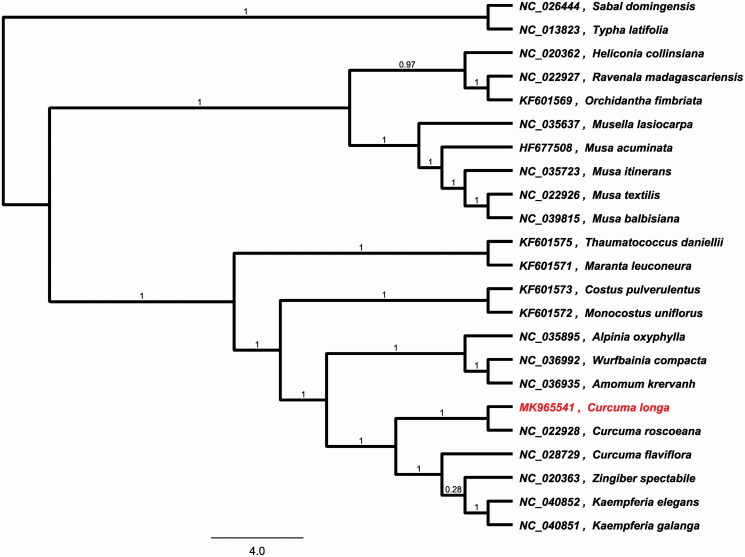
Phylogenetic tree inferred using maximum-likelihood (ML) method based on the complete chloroplast genome of 23 representative species. *Sabal domingensis* and *Typha latifolia* were used as the outgroup. A total of 1000 bootstrap replicates were computed and the bootstrap support values are shown at the branches. Genbank accession numbers were shown.
